# Acoustic Properties of Innovative Concretes: A Review

**DOI:** 10.3390/ma14020398

**Published:** 2021-01-14

**Authors:** Roman Fediuk, Mugahed Amran, Nikolai Vatin, Yuriy Vasilev, Valery Lesovik, Togay Ozbakkaloglu

**Affiliations:** 1 Polytechnic Institute, Far Eastern Federal University, 690922 Vladivostok, Russia; roman44@yandex.ru; 2 Department of Civil Engineering, College of Engineering, Prince Sattam Bin Abdulaziz University, Alkharj 11942, Saudi Arabia; 3 Department of Civil Engineering, Faculty of Engineering and IT, Amran University, Amran 9677, Yemen; 4 Peter the Great St. Petersburg Polytechnic University, 195251 St. Petersburg, Russia; vatin@mail.ru; 5 Department of Road-Building Materials, Moscow Automobile and Road Construction University, 125319 Moscow, Russia; yu.vasilev@madi.ru; 6 Belgorod State Technological University named after V. G. Shoukhov, 308012 Belgorod, Russia; naukavs@mail.ru; 7 Research Institute of Building Physics, The Russian Academy of Architecture and Building Sciences, 127238 Moscow, Russia; 8 Ingram School of Engineering, Texas State University, San Marcos, TX 78667, USA; togay.oz@txstate.edu

**Keywords:** concrete, noise, acoustic properties, sound-absorbing, sound-reflecting

## Abstract

Concrete is the most common building material; therefore, when designing structures, it is obligatory to consider all structural parameters and design characteristics such as acoustic properties. In particular, this is to ensure comfortable living conditions for people in residential premises, including acoustic comfort. Different types of concrete behave differently as a sound conductor; especially dense mixtures are superior sound reflectors, and light ones are sound absorbers. It is found that the level of sound reflection in modified concrete is highly dependent on the type of aggregates, size and distribution of pores, and changes in concrete mix design constituents. The sound absorption of acoustic insulation concrete (AIC) can be improved by forming open pores in concrete matrices by either using a porous aggregate or foam agent. To this end, this article reviews the noise and sound transmission in buildings, types of acoustic insulating materials, and the AIC properties. This literature study also provides a critical review on the type of concretes, the acoustic insulation of buildings and their components, the assessment of sound insulation of structures, as well as synopsizes the research development trends to generate comprehensive insights into the potential applications of AIC as applicable material to mitigate noise pollution for increase productivity, health, and well-being.

## 1. Introduction

**Figure 1:**
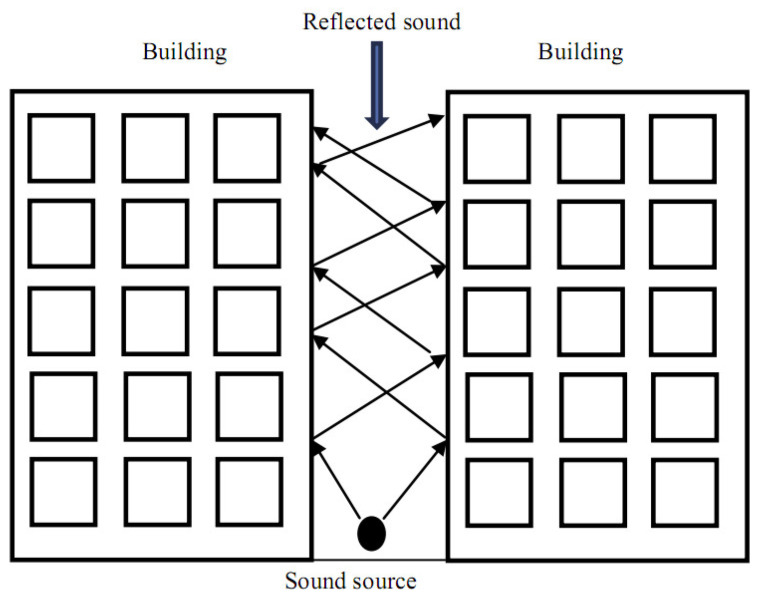
Sound waves reflections in a surrounded narrow thoroughfare [3]. Reprinted with permission from Elsevier [3].

Building regulations and planning authorities have not properly focused on resolving the ongoing issue of noise pollution faced by the residents of urban areas [1]. Typically, concrete is used as an external cladding to inhibit the propagation of sound transmission, which reflects the sound waves away from the structure [2]. Although the sound waves get reflected away, its magnitude does not reduce and becomes an issue in enclosed spaces such as apartment complexes, factories, and narrow thoroughfares, respectively (see Figure 1) [3,4]. As a result, this leads to several problems such as masked warning signals, higher chances of impaired hearing, and increased work-related stresses [5].

Concrete is a widespread and cheap structural material for the construction of residential buildings of any volume and number of storeys [6]. During the construction and operation of these buildings, it is necessary to ensure comfortable living conditions for people [7]. The most important step along this path is ensuring the acoustic well-being of residents through the optimal design of building envelopes [8,9,10]. Usually, when a sound wave strikes or hits the surface of a building, the sound energy gets absorbed, transmitted, or reflected away [11]. Hence, there is a relationship between the incident energy and the absorbed, transmitted, or reflected energy [12]. The absorption of sound in a porous material is related to the loss in energy wherein the incident noise energy converts to heat energy and some other energies due to vibration, friction, and air viscosity [13,14]. Sound insulation is the sound transmission loss of the building partition [15,16]. Lightweight porous materials absorb sound well, while heavyweight and hard materials reflect a significant portion of sound energy [3]. Despite the fact that there are some studies on the acoustic behavior of different concrete material, it is necessary to systematize the current state of sound-insulation concrete composites and structures made of them [17,18].

To reduce build time, labor savings, and thermal efficiency, most architects and designers prefer the use of insulated concrete forms. However, the acoustic properties of these insulated concrete forms are often overlooked or under looked. To provide better acoustic properties, the use of acoustic insulation concrete (AIC) is a recent advancement in the field of concrete technology. The acoustic component installed in an AIC reduces the sound in the building structure and keeps the pollution created by noise at bay while improving the acoustical effects of the sound generated within. To achieve a desired acoustic protection, the installation of an AIC wall can provide excellent exterior insulation as well as a soundproofing envelope. Due to a better soundproofing envelope, AIC becomes a suitable choice for hotels, airports, and schools where the requirement of thick traditional walls can be eliminated. Meanwhile, the use of lightweight concretes such as foam or fabric are usually porous and do not allow sound to be reflected. As a result, the sound passes through the porous medium, and the energy gets converted to heat while reducing its magnitude [3]. Hence, the use of lightweight concrete is effective when used in movie halls or in recording studios. This reduces the resonance time in the room [19,20]. Although the use of lightweight concrete is effective internally, it is not suitable in external conditions, and hence, concrete is still the preferred material for other commercial and residential building structures. Based on the past researchers, it was reported that various types of concrete behave differently as a sound conductor; especially dense mixtures are superior sound reflectors, and light ones are sound absorbers. It is found that the level of sound reflection in modified concrete depends on the type of aggregates, size, and distribution of pores and changes in concrete mix design constituents. The sound absorption of AIC is enhanced by forming open pores in concrete matrices by either using a porous aggregate or foam agent. To this end, this article reviews the noise and sound transmission in buildings, types of acoustic insulating materials, and the acoustic properties. This literature study also delivers a comprehensive review on the type of concretes, the acoustic insulation of buildings and their components, the assessment of sound insulation of structures, as well as to synopsize the research development trends to generate comprehensive insights into the potential applications of AIC as applicable material to mitigate noise pollution for increase productivity, health, and well-being.

## 2. Transmission of Noise and Sound in Buildings

**Figure 2:**
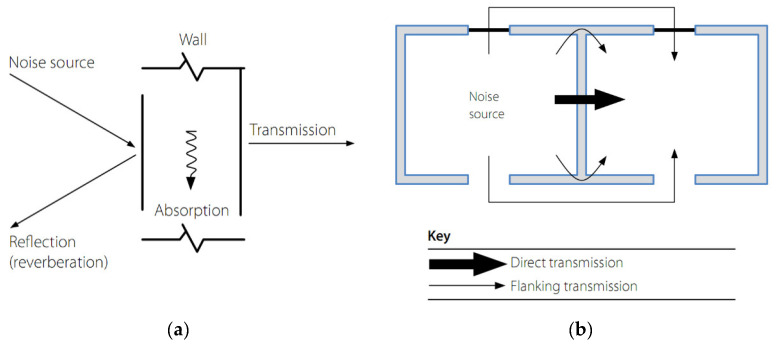
Noise and sound transmission in a building. (**a**) Factors of acoustic insulation performance; (**b**) Flanking and direct transmissions of sound between different zones [21]. Reprinted with permission from DATA STEEL [21].

To produce a noise and sound-proof AIC, the walls and roofs of a building could be engineered with modifications on the level at which the sound energy (Figure 2a) [21]:

- Reflects back from the building wall,
- Absorbs within the wall, and
- Transmits through the building wall.

Structural elements such as glazing and roof lights, which can reduce the performance, should also be considered. The transmission of noise between different zones of a building structure is shown in Figure 2b. Two types of noise transmission occur: direct and flanking transmission, and noise is transmitted when any two areas are separated from each other. The noise travels in two separate routes [22,23]: (i) through the separation structure, which is termed direct transmission, and (ii) around the separation structure through adjoining structural elements, which is termed flanking transmission [21,24]. Meanwhile, the insulation of sound for both types of transmission is controlled by its mass, isolation, and sealing characteristics [22,25]. The direct transmission of noise can be determined by laboratory tests, since it directly depends on the properties of the separation wall and floor [26]. Meanwhile, the details of junctions between the structural elements make it challenging to predict the flanking transmission of noise [23,27,28].

It should be noted that at various conditions, the separation wall could be of high standard AIC, while the side wall could be of low standard, which is continuous between the rooms. In such cases, flanking transmission can propagate more noise than direct transmission [21]. Hence, the joints between the separation elements should be properly detailed to minimize noise due to flanking transmission.

## 3. AIC Properties

The ability to reduce sound transmission through a structural element is what defines the properties of AIC. Usually, concrete is a good insulator of sound, which can reflect up to 99% of its total energy. However, it is also a relatively poor absorber of sound and can propagate within enclosed spaces, causing echoes [29,30,31,32]. Sound, a form of energy, can travel through mediums of solid, liquid, and gas in longitudinal wave by the oscillation of vibrating particles [1,33]. As the sound waves dissipate, it gets expanded outward while distributing the intensity over a larger area. The larger the vibrating particles in the medium, the more the energy that passes through the medium [34]. Meanwhile, audible sounds are classified into two types: airborne and impact sounds. Airborne sounds can be recognized as speech, loudspeakers, and musical instruments, respectively. These sounds waves travels through the air only and cannot travel through solids [3]. The airborne sound produces vibration in the concrete, which causes the vibration of suspended particles on the other side of the wall, thus, causing it to be heard. On the other hand, footsteps, closing doors, and falling objects are categorized as impact sounds, which can vibrate between walls and floors, leading to airborne noise in adjoining rooms [2]. Typically, noise and sound differ from each other, with the former being subjective and dependent on the receptor. Due to it being subjective, architects and designers should consider reducing noise in the building, in urban areas in particular [35]. However, since it is challenging to lower the volume or the propagation of sound, noise mitigation measures should be implemented to bring down the level of annoyance [36,37]. In this regard, remedial measures such as insulation, reflection, and isolation provide the best ways to reflect sound energy [3,26].

## 4. Types of Acoustic Insulating Materials

**Figure 3:**
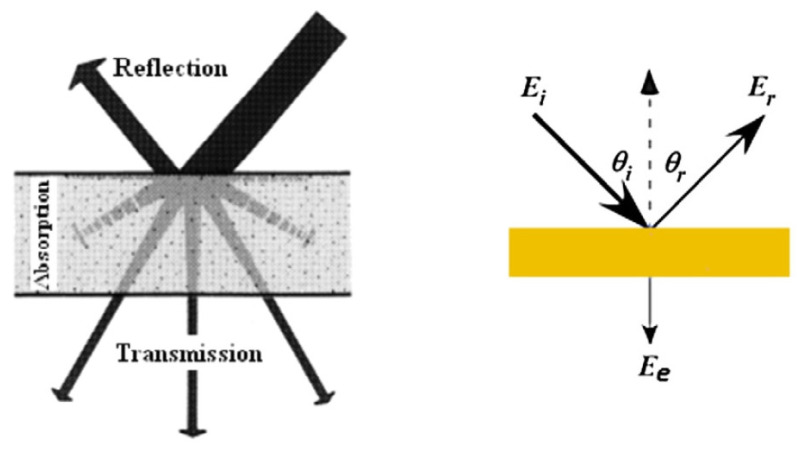
Sound energy conservation [14]. Reprinted with permission from Elsevier [14].

The common aspects concerning the conservation of acoustic energy according to the classic ratio (1) are shown in Figure 3.

*E*_*i*_ = *E*_*r*_ + *E*_*e*_ (1)

where *E_i_*—energy of sound falling on the building envelope; *E_r_*—reflected sound energy; and *E_e_*—comprises the absorption and transmission of sound energy.

For the design of enclosing structures, it is important to use extremely sound-absorbing or extremely sound-reflecting materials.

### 4.1. Sound-Absorbing

The acoustic absorption coefficient α is computed using Equation (1) is expressed as (2).

(2) $$\alpha =1-\frac{E_{r}}{E_{i}}$$

The absorption coefficient varies between 0 and 1, where 0 denotes the materials that reflect sound, and 1 is an ideal sound absorption material [38,39,40]. The presentation of the acoustic absorption coefficient as one value is an intricate problem.

**Figure 4:**
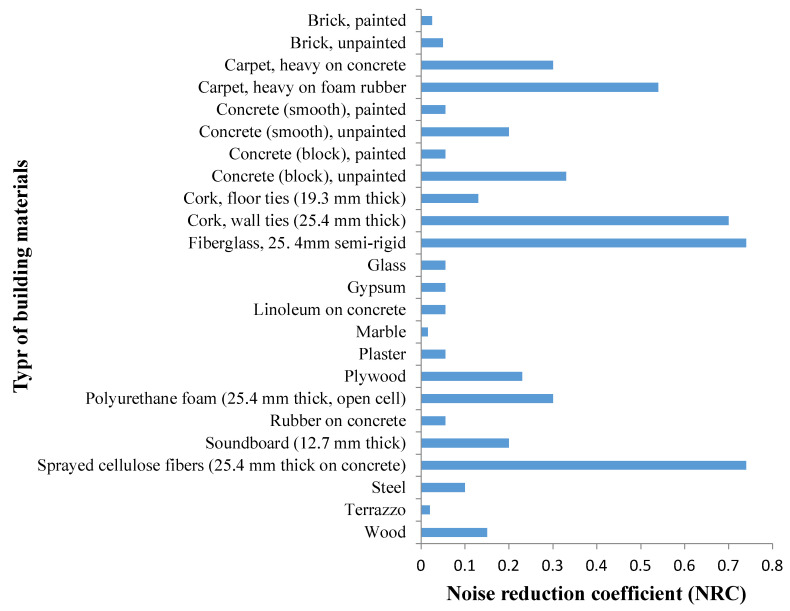
Building materials versus noise reduction coefficient (NRC) [43]. Modified with improvements from [43].

This is attributable to the fact that the coefficient of acoustic absorption, α, is varied for each frequency of sound. As a simplification, the division of the audible frequency spectrum into octaves (with a further 1/3 octave division) is used, as well as, for example, such characteristics as noise reduction coefficient (NRC) and sound absorption average (SAA) [8,16,41]. NRC is a part of the easiest approaches that could be estimated as the arithmetic mean of sound absorption coefficient at frequency ranging between 250 and 2000 Hz. Meanwhile, SAA is the arithmetic mean of the coefficients of sound absorption at 12 one-third octave frequency ranging 200–2500 Hz in conformity with ASTM C423-17 [42]. Construction materials are commonly ranked by their NRC [42,43] (Figure 4).

Summary of the noise reduction coefficients for different concrete composites are presented in Table 1. It can be concluded that an acoustic insulation concrete (AIC) building structure should possess a relatively good absorption coefficient with the NRC exceeding 0.45.

### 4.2. Sound-Insulating

Sound reflectors are materials that reflect noise, preventing the further propagation of a sound wave. Concrete, glass, wood, plastic, and metal are used as sound-reflecting materials.

In the concept of “Sound reduction index”, two quantities should be distinguished [47]:

- R_w_—sound insulation excluding flanking structures such as longitudinal walls, ceilings, floors, i.e., it is a value obtained during research in a laboratory;
- R’_w_—sound insulation of the structure, taking into account the flanking elements, i.e., sound transmission along longitudinal side walls, floor slabs.

The last parameter is more adequate; therefore, it is the one that is important for assessing sound insulation. It varies widely for different types of concrete. For example, rubber concrete with a smooth surface has this value at the level of 30–35 dB [54], and for concrete with crumb rubber, it is 30–50 dB [3]. Moreover, for hemp concrete, it fluctuates in the range of 3–11 dB [56], and for homogeneous concrete floors, this value reaches 60–70 dB, and theoretically, it can reach 90 dB [63].

The same indicator is “Transmission Loss (TL)”, which differs depending on the type of material. However, the sound reduction index R is defined by ISO standards, so, in general, it is usually preferred in the American literature. In addition, ISO 717-1 distinguishes between frequency-dependent R (or R’) and weighted values (R_w_ or R’_w_). The latter values are accepted for frequencies around 500 Hz; therefore, they are considered the most presentable, and the fact is that a significant part of the sound (noise) spectrum is concentrated in the region of 500 Hz. Accordingly, ISO 717-1 requires the weighted Rw (or R’w) value precisely in the frequency range of about 500 Hz. Thus, this is a somewhat simplified, but sufficient for practical application, interpretation for sound reduction index R (or R’). For the sake of clarity, soundproof building materials are commonly designed in such a way as to have a maximum sound absorption coefficient, specifically for noise with frequencies in the region of 500 Hz.

The efficiency of sound reflectors is evaluated by the following features of materials and structures:

- The surface weight of the cladding [64,65]. In building acoustics, there is a well-known “law of mass”, according to which doubling the mass of a single-layer fence leads to an increase in sound insulation. This works even more efficiently if the mass is concentrated in the surface layer, as can be seen from [66]. In addition, with an increase in the mass of the cladding, the resonant frequency of the system decreases, this also increases the sound insulation.
- The tightness of the structure. Slots and holes noticeably reduce the sound-insulating ability of the structure due to sound diffraction. For example, if a through hole of 2 × 2 cm in size is made in a 15 m^2^ partition, then the sound insulation of the partition will decrease by 20 dB [67].
- The presence of a sound absorber inside the frame allows for a multilevel dissipation of sound energy [68]. Thanks to such measures, resonances in the airspace become impossible.
- The depth of the cladding frame. With the distance of the cladding from the wall, the sound insulation grows. This is due to the fact that the resonance frequency of the structure with which the claddings begin to effectively perform sound-reflecting functions is reduced. For example, when doubling the air gap of the tested structure, the increase in sound insulation is reported without increasing the cost of the structure [69,70].
- The absence or minimization of rigid connections, for example, places of fixtures, which are the bridges of sound [34,71].

## 5. Type of Concretes

Table 2 shows acoustic property of different type of concretes. It can be seen that normal concrete detects the highest level of sound reflection followed by glass-based concrete.

### 5.1. Normal Concrete

The acoustic absorption coefficient for normal concrete is almost about 0.02, signifying that around 98% of the sound dynamism is a surface reflection. The more denser/heavy concrete, the higher the sound insulation rate that can be detected [3,18].

### 5.2. Aerated Concrete

In aerated concrete, alumina reacts with calcium hydroxide and leads to the appearance of gaseous hydrogen, which later forms microscopic bubbles in the mix. Lightweight aerated concrete has a density typically ranging 250 to 500 kg/m^3^ and has a higher porosity of about 82.1% to 91.5% [72]. The pore wall of this concrete is very thin due to which sound waves can be transmitted very easily. According to Laukaitis and Fiks [74], the porosity decreases as the density increases, but on the other hand, the volume of open pores also increases. The open pore volume of aerated concrete is more than that of foam concrete. The absorption coefficient of autoclaved aerated concrete is best categorized by the ratio of open pores to total porosity.

### 5.3. Foam and Porous Concretes

**Figure 5:**
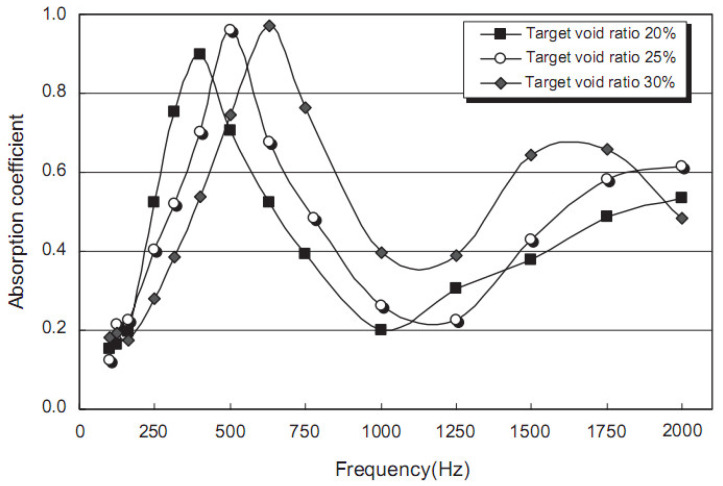
Sound absorption coefficient for the porous concrete with different densities and target void ratios (TVRs) [79]. Reprinted with permission from Elsevier [79].

Zhang et al. [51] developed a synthesis geopolymeric foamed concrete (FC) with absorption coefficients of 0.7 to 1.0 at 40–150 Hz and 0.1 to 0.3 at 800–1600 Hz, and 12 MPa of compressive strength was almost attained. Luna-Galliano et al. [75] studied the coefficient of absorption curves of geopolymers with silica fume used as a porous former. For different proportions of the starting components, the coefficient of sound absorption curves was the same, including with two peaks at 400 and 2500 Hz. It was noted that the mix contained more silica fume, which leads to the curve becoming wider (which indicates the stability of the coefficient of sound absorption), which can correlate with the highest open porosity. It was also found that an FC with low density exhibits a higher sound absorption coefficient (α) than a high-density FC when determined using a standing wave apparatus [76,77,78]. In Figure 5 [79], it can be observed that at the target void ratio (TVR) of 20%, the absorption coefficient revealed the highest value in the frequency range of 315–400 Hz; for the TVR of 25%, it ranked the second highest in the frequency range of 400–500 Hz; and for the TVR of 30%, it became the highest in the frequency range of 500–630 Hz [79]. This indicates that the frequency of the highest absorption coefficient was found for sound as the TVR augmented.

**Figure 6:**
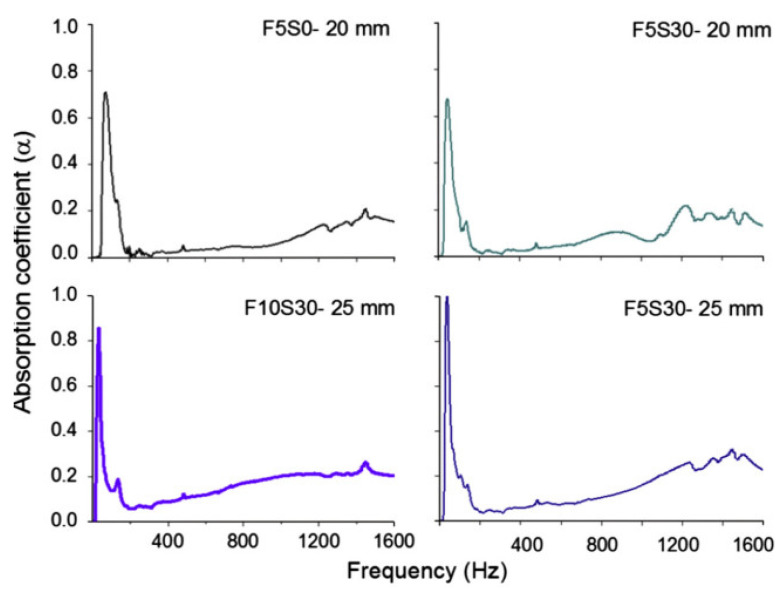
Acoustic absorption spectra of geopolymer foamed concrete (GFCs) at varying thickness [51]. Reprinted with permission from Elsevier [51].

In another study, it was found that the inclusions of 30% slag and fly ash into geopolymer FC (GFC) show no significant effect on the absorption coefficient at lower frequency but increases at higher frequency, 800–1600 Hz in particular [51,80]. This can be explained by the variation in the pore size, porosity, and tortuosity owing to inclusions of the slag particles [81]. Even increasing the dosage of foam (5 to 10%) shows little or no effect on the absorption coefficient at lower frequency but is efficient at medium frequency only, i.e., about 600–1000 Hz. Nevertheless, in comparison to a conventional plain concrete, which has an absorption coefficient typically less than 0.1 at 125–2000 Hz, GFC shows excellent acoustic absorption properties [82]. However, GFC has low sound absorption at medium to high frequencies relative to expanded clay porous concrete, which usually has an absorption coefficient of about 0.5 in this region [83]. However, a GFC shows the same absorption coefficient as the thickness of the GFC increases (Figure 6).

### 5.4. Crumb Rubber Concrete

Crumb rubber concrete (CRC), which is produced using different sizes of crumb rubber typically ranging 6 to 19 mm for coarse and 1 to 6 mm for fine, has been a subject of recent research [3,31,32,62,84]. However, the use of CRC in structural applications is not practical owing to considerable reduction in the strength [85,86]. Although CRC has lower mechanical strength, it can be used as a durable composite material capable of reflecting and absorbing sound [87]. In fact, the use of CRC as an exterior surface of a building can reduce the propagation of street noises into the buildings. High noise levels caused by busy streets and passing into dwelling spaces are often uncomfortable for inhabitants in high-rise apartments. An extremely dense material has an absorption coefficient of about 0 and can reflect away the sound completely. Hence, the use of CRC can provide a solution to both sound absorption and recycling used tires. Table 3 presents a list of classic absorption coefficients for some common materials used in structural concretes.

Materials with good sound absorption prepared by a concrete matrix incorporated with crumb rubber were investigated in [56]. The sound absorption coefficient α was above 0.5 for nine out of 12 specimens and attained a maximum value of 0.82 and 0.93 under favorable conditions. The concrete panels were prepared with crumb rubber as a replacement to natural fibers and investigated its acoustic properties in references [3,88]. The results showed that rubber concrete has proven itself in terms of sound absorption, especially with a high percentage (15%) of rubber crumb. Sukontasukkul [45] reported an improvement of 46% in the NRC upon the inclusion of 20% crumb rubber in precast concrete panels.

**Figure 7:**
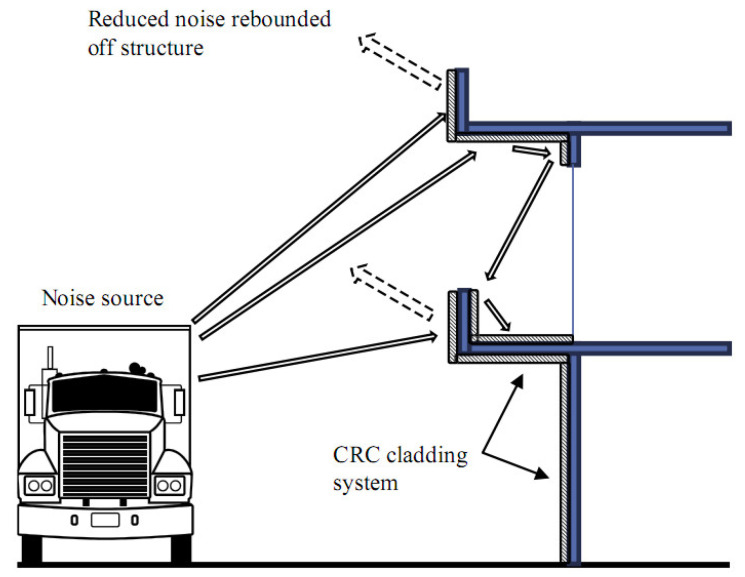
A crumb rubber concrete (CRC) cladding installed on a multi-storey structure to avoid the diffusion of sound [95]. Modified with improvement from *Wakefield* [95].

Materials containing crumb rubber have been known to be a good absorber of sound, with absorption coefficients ranging between 0.3 and 0.7 [32]. In fact, the combination of crumb rubber and concrete further can increase the absorption coefficient as well as reduce the level of reflected sound [89]. Apart from structural concrete, the use of crumb rubber has also been known to be a good sound absorber when utilized in highways. The incorporation of crumb rubber in asphalt mixes could lower the noise produced by vehicular traffic. The sound absorption coefficients of asphalt pavements containing crumb rubber are considerably improved with time due to its higher absorption energy [46]. The damping vibration was about 230% higher when crumb rubber was used to replace 15% of natural fines in CRC [90]. Incorporating 20% crumb rubber in concrete blocks was also reported to produce a lighter, flexible, and durable sound-absorbing material [91]. The durability of CRC against freeze–thaw could also be improved using air entraining admixtures [92,93]. It is found that CRC when exposed to excessive heat shows a substantial enhancement in the energy absorption, but only when lower grades of rubber and natural fines substitution were considered [85]. In comparison to conventional aggregates, crumb rubber is highly elastic, which deforms easily under an applied load, and its ductility can increase up to 90%, subsequently improving energy dissipation [3]. As the crumb rubber content increases, the density of CRC reduces. Since a material’s acoustic properties depend mostly on its density, lightweight concrete such as CRC containing a higher proportion of crumb rubber could absorb more sound. Hence, the ability to reflect more sound energy increases [32,94]. Due to this, CRC panels have been used mostly in office buildings as exterior cladding or on balconies of buildings. The compaction degree of a CRC also greatly influences its sound absorption, since the larger grades of CRC absorb more sound when fully compacted [62,90]. Meanwhile, CRC cladding panes are often used as an alternative to the conventional ones to protect the structure with an added benefit of reducing the overall weight. A proposal provided by the City of Vancouver Noise Control Manual [95] shows that the installation of exterior CRC panels around the balconies of high-rise buildings can improve its acoustic performance with a reduction in the level of reflected noise as well (as see in Figure 7).

In reference [96], the importance of preliminary processing of the surface of secondary rubber particles to increase the sound-insulating properties of CRC was proved. The elastic modulus of concrete combinations was determined, and the surface adhesion prepared from pretreated/untreated rubber aggregate was studied [3]. Conclusions indicated that the method of pretreatment caused weaker adhesion between the cement paste and crumb rubber; therefore, it improved the ability to absorb the vibration and sound-insulating properties of concrete. It was shown that the effects of freezing and thawing do not significantly affect the insulating properties [3]. It was been found that the insulation characteristics for all concrete improved at high frequencies. The findings show that rubber concrete can be used on the outside of building envelopes to absorb sound throughout multi-storey urbanized buildings, but it requires full-scale on-site testing.

### 5.5. Expanded Polystyrene Concrete

The use of foamed polystyrene aggregates has some effect on noise reduction owing to its lower stiffness, which has a high ability to dissipate energy. Expanded polystyrene granules (EPS) can easily be added in concrete to produce a lightweight concrete of varying density [60]. EPS are ultra-lightweight aggregates with a density <33 kg/m^3^, non-absorbent, hydrophobic, and closed cell nature [97]. The NRC of a 50 mm-thick layer of EPS is around 0.32 [98]. This means that about 32% of the incident sound energy gets absorbed and does not reflect back, hence improving the absorption characteristics of the material. Oancea et al. [60] replaced the aggregate with 50% polystyrene foam while obtaining lightweight concrete with a density of 1810 kg / m^3^ and NRC at the level of 0.18. Due to the closed porosity of polystyrene beads, effective damping of the sound wave is not possible.

### 5.6. Fibered Concrete

**Figure 8:**
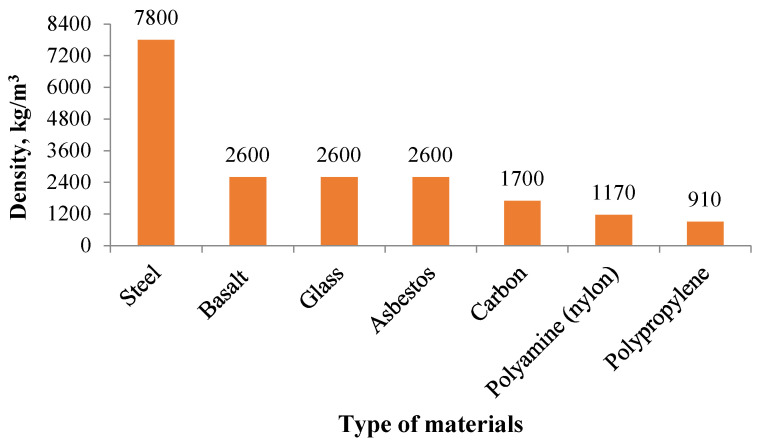
Densities of materials of various fibers [101,102]. Data adopted with permission from Fediuk and Klyuev [101,102].

At present, synthetic fibers such as glass and mineral fibers are used in buildings as a sound-proofing material [99]; however, these pose risks to human health [100]. It is also found that the airflow resistivity is an important parameter that needs to be considered in the acoustic behavior of fibrous concrete [12]. When choosing raw materials for concrete production, it is necessary to select materials with a reduced density that is capable of damping the sound wave. In particular, when choosing fiber, polypropylene has one of the lowest densities (Figure 8).

Stainless steel, nickel fiber, alloy fiber, and iron metallic fiber are some of the noise reduction materials available [12]. A review of existing literature on the acoustic absorption of metallic and inorganic fibrous composites is presented in Table 4. The use of fibers is widely known to provide a wide and varied solution to noise reduction in buildings, some of which are listed in Table 4.

### 5.7. Recycled Aggregate Concrete (RCA)

The sound absorption of a cellular material is mostly influenced by the energy loss occurred due to the friction induced within the pores of the walls [79]. Hence, the higher the open void ratio, the greater the SAC of concrete at all frequencies [103]. In a porous concrete, the pores are interconnected to each other, and the pores increase as the amount of coarse aggregate increases [104]. Consequently, the sound absorption of a porous materials is significantly influenced, which is directly proportional to its airflow resistivity [105]. Hence, to produce a sound-absorbing concrete, it is imperative to generate a tortuous path wherein the sound energies could dissipate, mostly in frequencies of noise traffic, ranging between 300 and 1000 Hz [106]. Some researchers used recycled aggregate with particle sizes greater than 5 mm as a substitute for aggregates in concretes [13,107].

**Figure 9:**
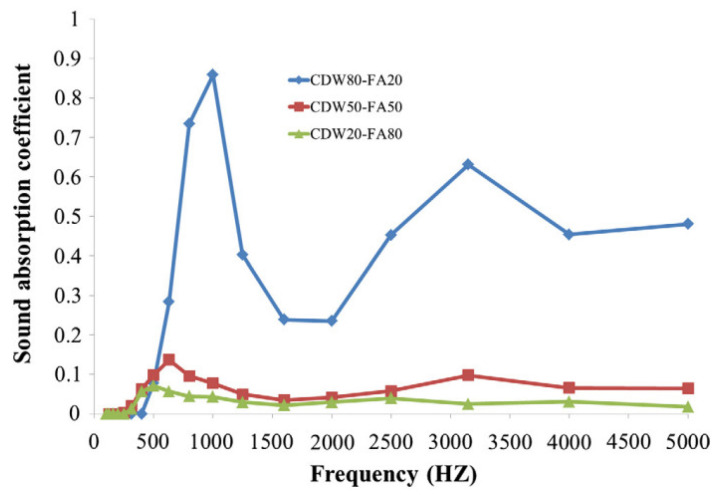
Sound absorption coefficient (SAC) for demolition wastes as aggregates for geopolymer concrete [13]. Reprinted with permission from Elsevier [13].

At the same time, the SAC was found to be enhanced with the use of recycled concrete aggregates [11]. An SAC of about 0.91 was recorded when 100% of RCA was used, which is 0.24 higher than the control mix [122]. This was as a result of the greater void content in the RCA concrete. Figure 9 [13] clearly portrays that the SAC increases as the demolition wastes aggregates (CDW)/fly ash (FA) increases. In fact, the porous concrete corresponding to the CDW80-FA20 mix showed the best SAC. Meanwhile, a huge variation can be observed when the amount of coarse fraction increases to 80%.

### 5.8. Mollusk Shell Waste Aggregate Concrete

It is also known that the particle size plays a crucial role in the acoustic properties of porous materials made with shell waste [49]. To examine this, the grain size distribution of the mollusk shell waste (7 and <−2 2 mm) was studied [41]. It was found that the porous concrete containing shell waste aggregates of about 5–10 mm showed the best acoustic absorption properties [49,79]. Meanwhile, it is reported that the internal pores have no contribution to the SAC of a porous concrete [55]. Hence, it was concluded that concretes made with mollusk shell waste (2 to 7 mm particle size) increase the SAC by about 40% with respect to the porous concrete containing natural aggregates only. [123]. For particles of the same size, the sound transmission lost showed absorption of more than 4 dB at a thickness of 12 cm. An improvement in the acoustic characteristics of the composites is possible due to the pyrolysis of mollusk shells, resulting in a convoluted surface capable of effectively damping the sound wave [73]. This finding coincides with the study by Paridah et al. [124] wherein the sound waves hitting the pores of a wall produce an acoustic wave due to the dissipation of energy. Due to the porosity and tortuosity in the specimen, an incident sound wave is produced, dissipated into sound energy due to internal friction, and results in high SAC [125]. Logically speaking, large particles cause a reduction in the energy dissipation due to its higher porosity, and hence, the absorption coefficient also decreases. Hence, sizes up to 7 mm are crushed and allowed to be utilized [41,49]. Owing to this, the SAC of a porous concrete is usually lower than a recycled concrete, mostly in frequencies below 2000 Hz [123].

### 5.9. Polymer Concrete

Arenas et al. [13] report that the activation solution in the geopolymerization of fly ash has a considerable effect mainly on the strength parameters but not on acoustic properties. However, according to Luna et al. [75], incorporating a higher amount of silica fume in the mix and increasing the setting temperature leads to an increase in the open voids. In turn, this reduces the compressive strength but increases the SAC. Similarly, Mastali et al. [59] developed cinderblock alkali-activated concrete with significant acoustic characteristics for the interior walls of buildings in residential areas. Stolz et al. [58] produced geopolymers with a density of about 1000 kg/m^3^ as well as heat and sound insulation comparable to the properties of the commercial existing options. In comparison to ordinary Portland cement (OPC)-based composites, alkali-activated FC shows excellent acoustic absorption characteristics [44]. Porous concrete was also found to exhibit average SACs higher than 0.5, at mid to high frequencies [83]. The alkali-activated FC was also observed to exhibit good SAC (>0.5) at the same frequency levels as that of the porous concrete [59].

### 5.10. Foam-Glass Based Concrete

**Figure 10:**
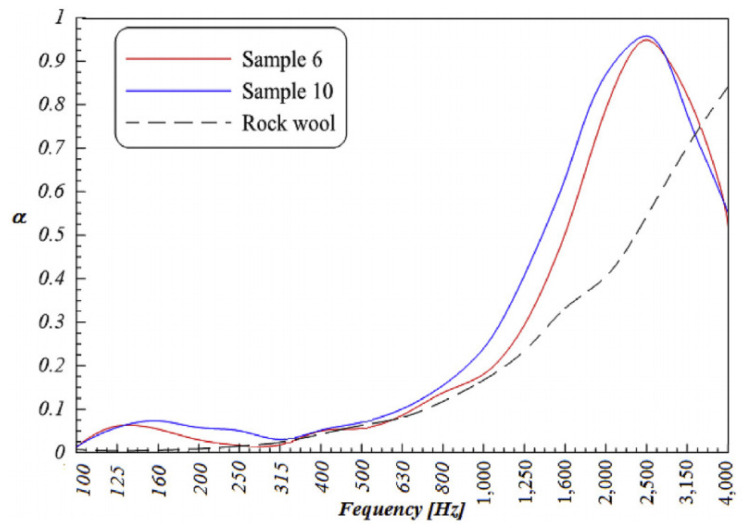
Average SACs of samples compared with rock wool [127]. Reprinted with permission from Elsevier [127].

The use of foam-glass concrete (FGC) can also provide a good acoustic insulation wherein the annealing step is not required, and the density can also be greater than 150 kg/m^3^ [126]. In fact, foamed-glass blocks of high costs can exhibit better aesthetics for indoor acoustic insulation properties [127]. However, an FGC composite is a unique material with good acoustic properties, with an almost unlimited service life [126,128,129,130,131]. The main reason for the insufficiently widespread use of foam glass in structures is not its operational features, but the cost related with the technological features of its manufacturing. Glass fiber is widely used in cement materials, which do not show a considerable improvement in sound absorption, but it improves sound reflection well due to the creation of a dense and low-porous structure [128,132,133,134,135]. Although significant studies have investigated FGC, very few characterized its open and closed porosity in detail [136,137,138]. The majority of the available research focused only on the partially closed, open porosity, closed porous with high density, or open porosity with lower-density FCs [139,140,141]. FGC with open porosity is usually due to the use of a decomposition foaming agent that crystallizes [139,140,141,142,143,144]. This type of concrete is produced using a replicate synthesis that was obtained from organic foams, the sol–gel process, or inorganic gel casting [127,145,146]. Meanwhile, only a few studies are available on FGCs with closed porosity and a density lower than 150 kg m^−3^ [139,147,148,149,150,151,152]. The SAC as a function of frequency containing 20% glass and 80% GDL (sample 6 and 10) has also been studied (Figure 10) [127]. From this study, it has been found that findings were insignificant at frequencies lower than 100 Hz. Meanwhile, a few researchers observed that the SAC of glass foam is higher than rock wool at medium to high frequencies [153,154,155]. This is mainly due to the porous nature of the material. Since foam is composed of an open cell structure, it shows excellent performance at a frequency range of 1250–3150 Hz [156]. Similarly, tortuosity also causes the SAC to vary while in transit from an open cell structure to a fibrous one [157]. In addition, due to the dimensional stability of FGC with open porosity, it is suitable as immovable acoustic insulation elements or as a provision for catalysts [145].

## 6. Building Components

Evaluation of the acoustic characteristics of the building elements allows you to deal with the noise penetrating the premises and determine the degree of its impact [158,159]. Building acoustics study the transmission of sound (for example, the steps of people and the noise of passing cars) through the walls and entrances of a building [158,160]. First, sound is measured inside and outside the building, and then work is carried out in the room to change the difference in the levels of reverberation and background noise [161,162]. Information about noise (for example, about its frequency spectrum) allows you to effectively deal with it, for example, using isolation and shielding [163,164].

### 6.1. Reinforced Concrete Wall System

**Figure 11:**
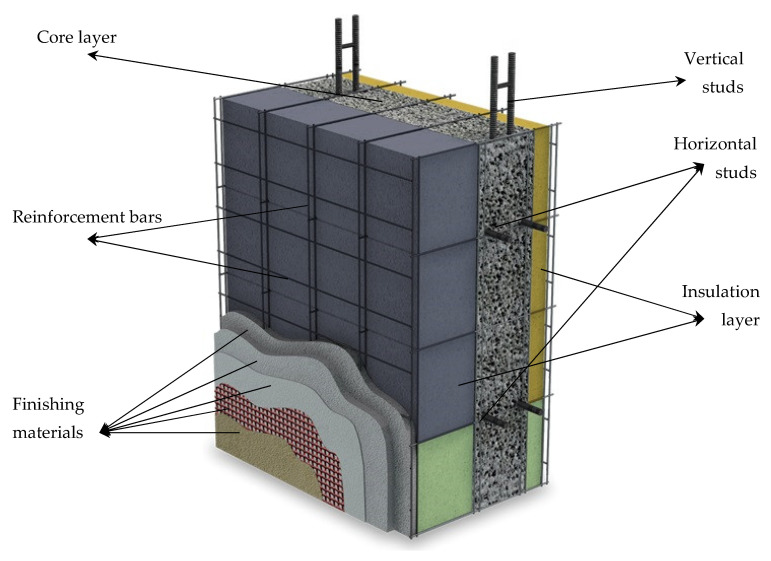
Model for the construction of reinforced concrete walls with extremely high acoustic insulation [167]. Reprinted with permission from ECOSISM [167].

A reinforced concrete wall has better sound-insulating properties than a brick of the same thickness, because it is denser [96]. A thick concrete wall is better than a thin concrete wall in terms of sound insulation [19]. However, in order for a single-layer reinforced concrete wall to provide standard sound insulation for urban conditions, it is necessary to ensure that its thickness is more than 1 m, which is very expensive and significantly reduces the internal space [165]. Therefore, the use of multilayer wall systems for building facades is very relevant [166]. Figure 11 shows a clear model for the construction of RC walls with extremely high acoustic insulation. Carefully selected layer thicknesses and their sequence ensure a sound reduction index of up to 60 dB [167].

### 6.2. Steel Plate Wall System

**Figure 12:**
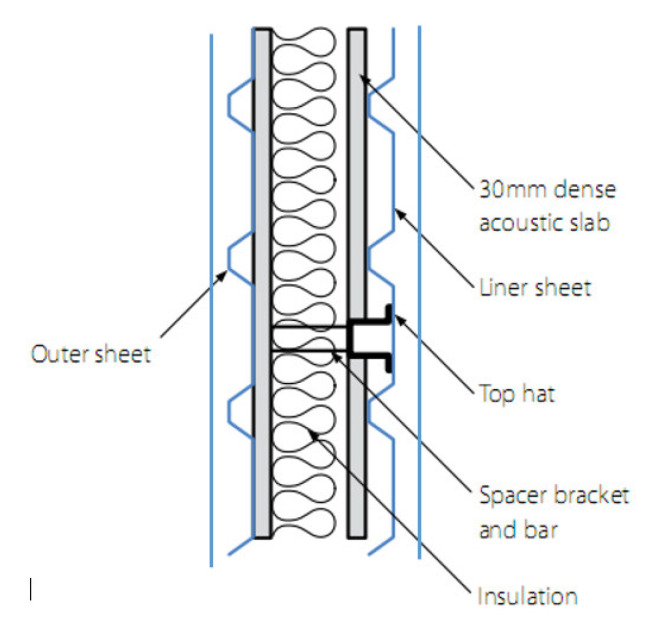
Effect of the of wall’s thickness built-up on acoustic insulation [21]. Reprinted with permission from DATA STEEL [21].

Good sound insulation properties are provided by wall systems using steel plates and an air inner layer, which are called ventilated facades [168]. The system of ventilated facades is not only an architectural solution, protection from adverse weather conditions, and reliable thermal insulation, but it is also an optimal sound-insulating property (Sound reduction index, R_w_) that can be improved up to 15 dB [169]. One of the widely used approaches in reducing airborne noise transmission is by the installation of a denser acoustic mineral wool wall, as shown in Figure 12 [21]. This dense mineral wool slab shows an excellent SAC and also acts as an additional layer to the structural concrete.

The insulation layer is usually installed in addition to the insulation quick, which maintains the low-level coupling between the two skins. It also maintains the thermal properties of the structural concrete. Meanwhile, the usage of a mineral wool acoustic wall can compromise with the acoustic performance due to its higher degree of coupling [98]. However, the additional mass provided by the acoustic mineral wool wall can reduce the impact noise propagated through the structure during rains [21].

### 6.3. Masonry Wall System

**Figure 13:**
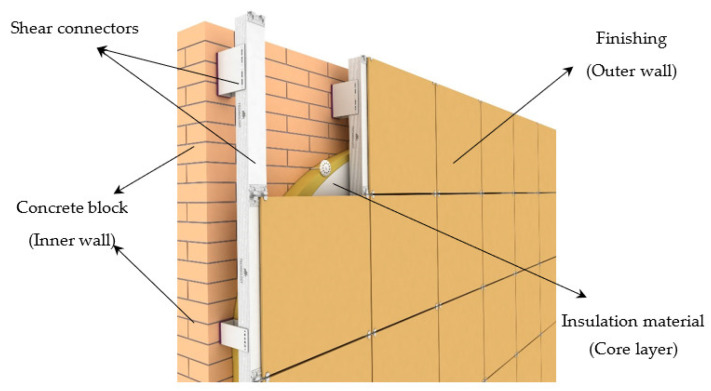
Masonry wall system [174]. Reprinted with permission from ORION-SD [174].

The compositional nature of the construction of the three-layer facade, combined with a brick wall, allows combining materials with excellent sound-absorbing and sound-reflecting properties (Figure 13) [170,171]. The mineral wool layer located inside has good sound absorption, which reduces the noise load by more than two times (TL and R_w_), especially from low-frequency sounds [172,173].

### 6.4. Concrete Sandwich Panels

A study by Frazao et al. [175] developed innovative structural panels, a combination of sisal fiber–cement composite as the thin outer layer, and a polypropylene-reinforced lightweight concrete as the base layer. Such multilayer composition makes it possible to efficiently perform sound-insulating functions by distributing sound-absorbing and sound-reflecting functions between the layers. Experimentally determined layer thicknesses and their sequence provide a sound reduction index R_w_ up to 50 dB [175]. Cuypers and Wastiels [176] made sandwich panels with outer layers of textile-reinforced concrete. Due to the fact that textile concrete is a light type of concrete, this allows for the creation of the effective sound absorption of a wave’s incident on the wall, both outside and inside. In this case, the sound reduction index R_w_ of the developed design is 45 dB. Good acoustic properties (R_w_ = 40 dB) are predicted for multilayer panels with corrugated steel surfaces and a core of plain or reinforced foam, as developed by Flores-Johnson and Li [177].

### 6.5. Reinforced Concrete Slab System

In urban apartment buildings, ensuring the sound insulation of floors between floors is of great importance [63,178]. To do this, it is necessary to lower the level of impact noise through the usage of various damping additives in the ceiling, for example, rubber [179]. The transmission of impact sound through hard-walled ceilings is greatly increased (R_w_ = 45 dB); therefore, rigid joints must be avoided [180,181].

**Figure 14:**
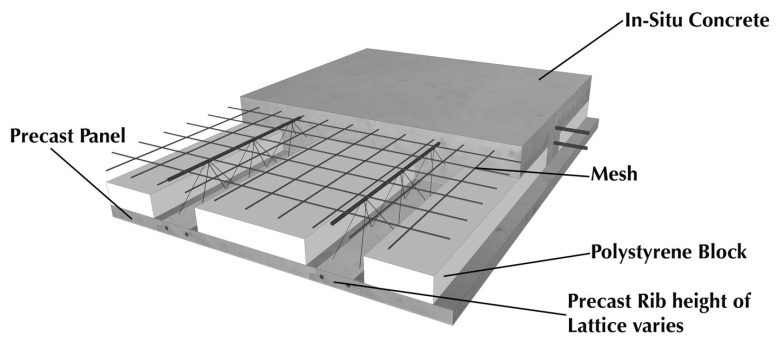
Details the Cobute precast slab system [185]. Reprinted with permission from Cobute [185].

Traditionally, hollow concrete slabs are utilized to lower the volume of concrete in the slab and to minimize its own weight; however, in this case, the resistance to impact noise is reduced (R_w_ = 36 dB) [182]. This is due to the fact that, unlike wall structures, the sounds of steps, moved furniture, etc., which are effectively extinguished by massive structures, are transmitted through the ceilings [183]. Al-Rubaye et al. [184] developed a hollow composite reinforcing system with four flanges with an aim to enhance the adhesion of concrete, stabilize holes in concrete elements, and improve sound insulation performance (R_w_ = 43 dB). It is also reported that the built-up system of Cobute precast slab (Figure 14) had a 50% reduction on self-weight, dramatically assisted with services distribution, and effectively contributed acoustic insulation (R_w_ = 40 dB) [185].

### 6.6. Steel–Concrete Composite Floorings

**Figure 15:**
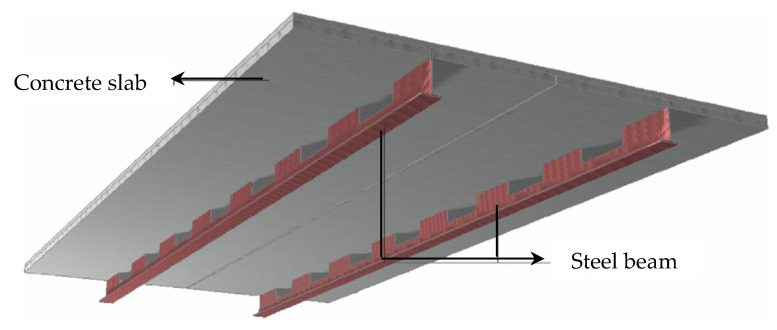
Steel–concrete composite floorings [186]. Reprinted with permission from Elsevier [186].

In comparison to reinforced concrete slabs, composite floor systems offer a more economical solution (Figure 15) [186]. The constituent structural elements of the reinforced concrete slabs can optimize the strength and performances of the steel and the concrete as well. However, due to the connection of the rigid system “reinforced concrete slab–steel beam”, impact and structural noise quite easily find their way to the lower floor. To improve the acoustic characteristics of the floors, various modifications of these composites have been developed, such as slim floor beams [187], Thor and Delta beams [188], composite slim floor beam [189,190], iTECH composite beams [191], and Ultra-Shallow Floor beams [192,193,194] are the other few composite flooring systems used. Some of these composite flooring systems use a profile steel sheet or small mesh beam [187,188,195]. This provides a better sound insulation to the floor (R_w_ = 30–50 dB).

## 7. Conclusions

Under the conditions of the current world development, noise pollution associated with population growth and industrial development is the main problem faced by people living mainly in municipal areas. This condition highlights the importance for long-term research of new energy materials that can reduce the acoustic power of a sound wave as a result of absorption and reflection. From the literature reviewed, it is found that each construction material has a different NRC and fundamentally relies on its density. However, to avoid sound waves, in any building materials, as far as possible, mass is required—that is, high-density materials, such as bricks, are best suited for partitions, while cellular concrete is lightweight and inferior to bricks in sound insulation of wave noise. However, it is imperative to note that regarding the structural noise of lower frequency, a cellular concrete isolates better than brick. The construction industries worldwide have started to mainly use the sound-absorbing concrete to reduce the level of sounds in opened and closed areas and increase the amount of sound insulation.

To increase sound insulation, wall structures should be designed as multilayer, including with the use of air gaps. Slab floorings that work as absorbers of structural noise should be designed using damping components, such as chopped rubber, and it is also necessary to prevent rigid “sound bridges”. It is proposed to expand the range of measured acoustic characteristics by expanding the lower boundary of the frequency range from 100 to 20 Hz.

However, it is obligatory to consider all structural parameters and design characteristics, when designing concrete structures, such as acoustic insulation property. This is, in particular, to ensure comfortable living conditions for people in residential premises, including acoustic comfort. Different types of concrete behave differently as sound conductors; especially dense mixtures are superior sound reflectors, and light ones are sound absorbers. It is found that the level of sound reflection in modified concrete highly relies on the type of aggregates, distribution and size of pores, and changes in concrete mix design constituents. The sound absorption of AIC can be improved by forming open pores in concrete matrices by either using a porous aggregate or foam agent. To this end, this article reviews the noise and sound transmission in buildings, types of acoustic insulating materials, and the acoustic properties. This literature study also provides a critical review on the type of concretes, the acoustic insulation of buildings and their components, and the assessment of sound insulation of structures, as well as synopsizes the research development trends to generate comprehensive insights into the potential applications of AIC as an applicable material to mitigate noise pollution for increase productivity, health, and well-being. Nevertheless, AIC is deemed as one of the superior materials that should be used in the building constructions, in particular, concert halls, cinemas, theatres, music venues, etc. Several further research investigations are recommended for the production of renewable and green concrete composite;

- New applications of AIC are worth exploring and can be found; for example, EPS-based concrete can be produced as a class of innovative lightweight soundproof concrete.
- To further study the potential use of modified concrete to develop high-sound insulation performance concrete.
- To increase the acoustic insulation performance of AIC in a hardened state using ecofriendly materials.
- To further extend the possible utilization of AIC in the building construction with a sound-insulated system and future sustainable cities with reduced noise and sound transmission.

## Figures and Tables

**Table 1:** A summary of NRC for different concrete composites.

Ref.	Year	Type of Specimen	Descriptions			Density (kg/m^3^)	α at Octave Frequency (Hz)				NRC
			Percentage (%)	Type of Materials	Size of LWA, mm (Thic. of Specimen, mm)		250	500	1000	2000	
[44]	2004	Concrete with LWA	20%	Lightweight cenospheres	10 to 300 μm (25 mm)	2310	0.04	0.10	0.21	0.17	0.13
			40%			2250	0.05	0.11	0.23	0.21	0.15
			60%			2180	0.05	0.10	0.11	0.12	0.11
[45]	2009		10%	CR	<4.75	2170	0.12	0.12	0.32	0.17	0.18
			20%			2110	0.11	0.10	0.37	0.15	0.18
[40]	2010		-	Expanded shale with 1.0% AE agent	4–8	1254	0.08	0.24	1.00	–	0.44
			-	Expanded shale with 1.0% AE agent	8–12	1189	0.06	0.22	0.96	–	0.41
[46]			10%	CR	<4.75	2200	0.05	0.10	0.06	0.07	0.07
			20%			2100	0.07	0.08	0.09	0.10	0.09
			30%			2000	0.12	0.13	0.11	–	0.12
[47]	2011	Hemp concrete	10, 15, 75%	Lime		1060	0.4	0.48	–	–	0.44
[48]	2012	Concrete with LWA	60%	Bottom ash	<10	701	0.21	0.24	0.28	0.28	0.25
[49]	2013	Mortar with LWA	80%		1–10 μm	1470	0.65	0.62	0.61	0.56	0.61
[50]	2014	Plaster with lightweight	80%	Insulation plaster and aerogel	−(10 mm)	300	0.03	0.08	0.06	–	0.06
[51]	2015	Alkali-activated cellular concrete	5%	Foam dosage	-	1050	0.05	0.10	0.15	–	0.10
			10%		-	960	0.06	0.10	0.20	–	0.12
[52]		Pervious concrete	1: 5	Vermiculite	0.5 to 4	640	0.10	0.21	0.78	0.32	0.35
[53]	2016	Hemp concrete	0.5% and 30%	30% GGBS and 0.5% ethyl cellulose	-	522	–	0.52	0.45	0.53	0.50
			30%	GGBS	-	505	–	0.49	0.42	0.44	0.45
			80%, 20%, and 0.5%	Hydrated lime, MK, and methyl cellulose	-	469	–	0.42	0.37	0.41	0.40
			80% and 20%	Hydrated lime, MK	-	493	–	0.46	0.39	0.44	0.43
[54]		Concrete with LWA	20%	CR	4–8	2264	0.06	–	–	–	0.05
			40%			2156	–	0.45	–	–	0.04
			60%			2026	–	0.54	–	–	0.06
			80%			1858	0.06	–	–	–	0.05
			20%	FCR		2313	–	0.30	–	–	0.05
			40%			2139	–	–	0.34	–	0.06
			60%			2032	–	–	0.43	–	0.10
			80%			1851	–	–	–	0.23	0.20
[55]	2017		10%	Miscanthus fibers	2–4	1504	0.06	0.20	0.25	0.07	0.15
			20%			1406	0.02	0.06	0.36	0.00	0.11
[13]		Geopolymer concrete with LWA	80%	C and D waste	<10	1510	0.00	0.06	0.85	0.23	0.29
[56]		Hemp concrete	-	Hemp shiv	5	590	0.13	0.28	0.91	0.48	0.45
[57]		Pervious concrete	50%	Arlite		700	0.08	0.12	0.46	0.23	0.22
[58]	2018	Alkali-activated cellular concrete	20–50%	Fly ash and 3:1 by mass. Adequate foam	-	940	–	0.25	–	–	0.19
						1130	–	0.23	–	–	0.24
						1310	–	0.18	–	–	0.11
[59]			35%	Foam dosage	-	600	0.20	0.10	0.40	0.94	0.41
			30%		-	720	0.18	0.16	0.54	0.78	0.42
			25%		-	820	0.03	0.12	0.43	0.85	0.36
[60]	2019	Concrete with LWA	50%	Polystyrene granules	1–4	1810	0.16	–	–	–	0.18
			50%	Polyethylene terephthalate	1–4	2047	0.22	–	–	–	0.18
			50%	Corn cob granules	size 1–6	1775	0.20	–	–	–	0.19
[61]		Hemp concrete	1:2	Hemp shiv	-	605	0.13	0.31	0.81	0.48	0.43
			1:2	Hemp fiber	-	407	0.19	0.63	0.83	0.71	0.59
[62]	2020	GGBS-based concrete	5–30%	GGBS as coarse and fine aggregates	1–4 and 4–8	419–995	-	0.54	-	-	0.24
[61]		Foam-glass concrete	92%	Foam bubbles	0.5–1.35	107–143	-	-	0.57	0.67	0.56

**Table 2:** Acoustic property of different type of concretes.

Type of Concretes	Maximum Coefficient of Sound Absorption	Level of Sound Reflection	Maximum Decrease in Sound Level at Frequencies, Hz	Refs.
Normal concrete	0.05–0.10	High	3000–5500	[52,57]
Aerated concrete	0.15–0.75	Low	250–2500	[44,72]
Foamed concrete	0.13–0.50	Low	100–2000	[51,61]
Crumb rubber concrete	0.30–0.70	Medium	400–2500	[3,54]
Polyurethane concrete	0.08–1.0	Low	150–1400	[55,60]
Coal bottom ash concrete	0.05–0.31	Medium	500–3500	[41,58]
Coconut fibers concrete	0.42–0.80	Medium	1250–3200	[59]
Recycled aggregate concrete	0.01–1.0	Medium	1500–2000	[60]
Oyster shell waste aggregate concrete	0.43–0.53	Low	1000–1800	[73]
Polymer concrete	0.90–1.0	Low	64–1600	[55]
Glass-based concrete	0.20–0.37	High	250–3150	[61]

**Table 3:** Sound absorption coefficients for some common materials.

Type of Material	SACs
Concrete	0.02–0.06
Hardwood	0.3
Unpainted block-work	0.02–0.05

**Table 4:** Acoustic insulation of different fibrous concrete composites.

Type of Fiber	Main Findings	Refs.
Rock wool	Similar acoustic behavior to glass wool	[108]
Carbon and glass fiber	Composites made with carbon fiber has higher SAC relative to glass fibered composite	[109]
Fibrous metal materials	Used to make silencers in cars	[110]
Glass wool	Comparison made between the Bies–Allard and Kino–Allard’s acoustic methods	[111]
Metal fiber felts	Used as an absorption material in silencers	[112]
Glass fiber-reinforced epoxy	Investigated the acoustic absorption properties of different composites	[113]
Sintered fibrous metals	Determined anisotropic acoustic properties of of sintered fibrous metals	[35]
Glass fiber recycled from deserted print circuit boards	Utilized for noise-reducing applications	[114]
Metal fiber	Absorption properties depends on the material properties such as the diameter, porosity, and thickness of fiber	[115]
Glass fiber felt	The direction of sound incidence and structure of the composite affects the sound insulation	[115]
Carbon fiber	Increases the sound absorption coefficient of a helical-shaped composite sound absorber	[116]
Fouled sintered fiber felts	Depends on the flow resistivity measurements	[117]
Basalt fiber	Panels shows a good absorption coefficient that increases with thickness and density	[118]
Carbon fiber	Composites made with carbon fibers shows higher absorption coefficients than Kevlar fiber at low to medium frequencies	[119]
Glass fiber-filled honeycomb sandwich panels	Improves the absorption coefficient at frequencies below 4.5 kHz	[120]
Metal fiber porous materials	Porous material can effectively enhance the sound absorption coefficient	[121]

## Data Availability

Data sharing not applicable.

## Abbreviations

AIC	Acoustic insulation concrete
CR	Crumb rubber
CRC	Crumb rubber concrete
EPS	Polystyrene granules
FA/GGBS	Fly ash/Ground granulated blast furnace slag
FC	Foam concrete
FCR	Fiber crumb rubber
FGC	Foam glass concrete
GDL	Brown algae, D-Gluconic acid δ-Lactone
GFC	Geopolymer foam concrete
ID	Identifier
LWA	Lightweight aggregate
LWC	Lightweight concrete
NRC	Noise reduction coefficient
OPC	Ordinary Portland cement
RC	Reinforced concrete
Rw	Sound reduction index
SAA	Sound absorption average
SAC	Sound absorption coefficients
TL	Transmission loss
